# Temporal stability of the hybrid zone between *Calocitta* magpie‐jays revealed through comparison of museum specimens and iNaturalist photos

**DOI:** 10.1002/ece3.9863

**Published:** 2023-03-15

**Authors:** Alana K. Pizarro, Devon A. DeRaad, John E. McCormack

**Affiliations:** ^1^ Moore Laboratory of Zoology Occidental College Los Angeles California USA; ^2^ Biodiversity Institute and Department of Ecology & Evolutionary Biology Kansas University Kansas Lawrence USA

**Keywords:** birds, gene flow, global change, habitat change, introgression

## Abstract

Hybrid zones are natural experiments for the study of avian evolution. Hybrid zones can be dynamic, moving as species adjust to new climates and habitats, with unknown implications for species and speciation. There are relatively few studies that have comparable modern and historic sampling to assess change in hybrid zone location and width over time, and those studies have generally found mixed results, with many hybrid zones showing change over time, but others showing stability. The white‐throated magpie‐jay (*Calocitta formosa*) and black‐throated magpie‐jay (*Calocitta colliei*) occur along the western coast of Mexico and Central America. The two species differ markedly in throat color and tail length, and prior observation suggests a narrow hybrid zone in southern Jalisco where individuals have mixed throat color. This study aims to assess the existence and temporal stability of this putative hybrid zone by comparing throat color between georeferenced historical museum specimens and modern photos from iNaturalist with precise locality information. Our results confirm the existence of a narrow hybrid zone in Jalisco, with modern throat scores gradually increasing from the parental ends of the cline toward the cline center in a sigmoidal curve characteristic of hybrid zones. Our temporal comparison suggests that the hybrid zone has not shifted its position between historical (pre‐1973) and modern (post‐2005) time periods—a surprising result given the grand scale of habitat change to the western Mexican lowlands during this time. An anomalous pocket of white‐throated individuals in the northern range of the black‐throated magpie‐jay hints at the possibility of prehistorical long‐distance introduction. Future genomic data will help disentangle the evolutionary history of these lineages and better characterize how secondary contact is affecting both the DNA and the phenotype of these species.

## Graphical abstract

We compare historical museum specimens to modern citizen science photos to test whether the hybrid zone between two species of magpie‐jays has changed over time. By scoring throat coloration across a geographic transect, we find that a narrow hybrid zone exists between the two species and that it has not changed substantially over time. Hybrid zone stability is notable in this system because of the dramatic habitat changes across the landscape of these two species in the lowlands of Western Mexico.

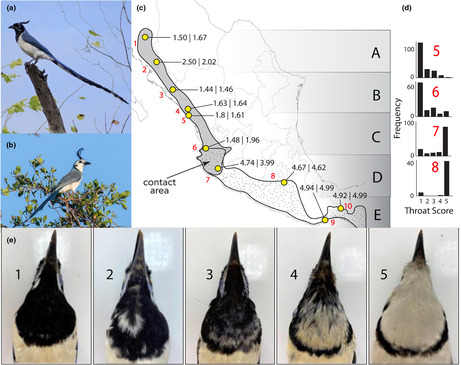

## 1 INTRODUCTION

An estimated 1%–10% of all species hybridize, making it a common phenomenon with widespread ramifications for ecology and evolution (Mallet, 2005). Many hybrid zones are described as tension zones, where dispersal of parental types to the center of the zone is balanced by selection against hybrids, resulting in a cline that eventually reaches a stable equilibrium (Barton & Hewitt, 1985). However, a growing body of research shows that hybrid zones can be dynamic, capable of moving and changing (Wielstra, 2019) due to differences in fitness between parental taxa (Dasmahapatra et al., 2002) or human‐mediated disturbances to habitat and climate (Ryan et al., 2018; Taylor et al., 2015). Continued documentation of the temporal dynamics of hybrid zones will further our understanding of how secondary contact affects speciation and how global change might affect the maintenance of biodiversity (Vallejo‐Marín & Hiscock, 2016).

Studying temporal changes in hybrid zones can be challenging. Time‐series data are needed at multiple sites along a transect, but the combination of adequate sampling over space and time is rare (Alexander et al., 2022; Cicero et al., 2021; Leaché et al., 2017). Modern follow‐up studies to some of the classic hybrid zones have provided valuable insights into the temporal dynamics of hybrid zones, documenting results ranging from temporal stability (Wang et al., 2019) to widening of a hybrid zone (Engebretsen et al., 2016), as well as shifts in location (Aguillon & Rohwer, 2022; Billerman et al., 2019; Carling & Zuckerberg, 2011; Smith et al., 2013; Walsh et al., 2020) or elevation (Morales‐Rozo et al., 2017). Continuing advances in data aggregation offer an opportunity to expand temporal comparisons of hybrid zones from opportunistic studies of historically well‐sampled clines to broader efforts. Specifically, the growth of online aggregates of photos (e.g., Google Images; Leighton et al., 2016) and citizen science platforms like iNaturalist offer crowd‐sourced, georeferenced voucher photos, which contain records of modern hybrid individuals (Minor et al., 2022) and potentially scorable hybrid traits (Fritz & Ihlow, 2022). Additionally, the continued aggregation of natural history collection databases (Hedrick et al., 2020; Peterson & Gordillo‐Martínez, 2002) means researchers can assemble densely sampled transects by combining specimens from multiple museums, often without the need to visit them thanks to continuing efforts toward photo‐digitization (Blagoderov et al., 2012; Medina et al., 2020). Combined, these data sources offer the potential for broad and dense temporal sampling to provide before‐and‐after snapshots of geographic variation. But, to our knowledge, these resources have not yet been used in tandem to assess temporal change in a hybrid zone.

**FIGURE 1:**
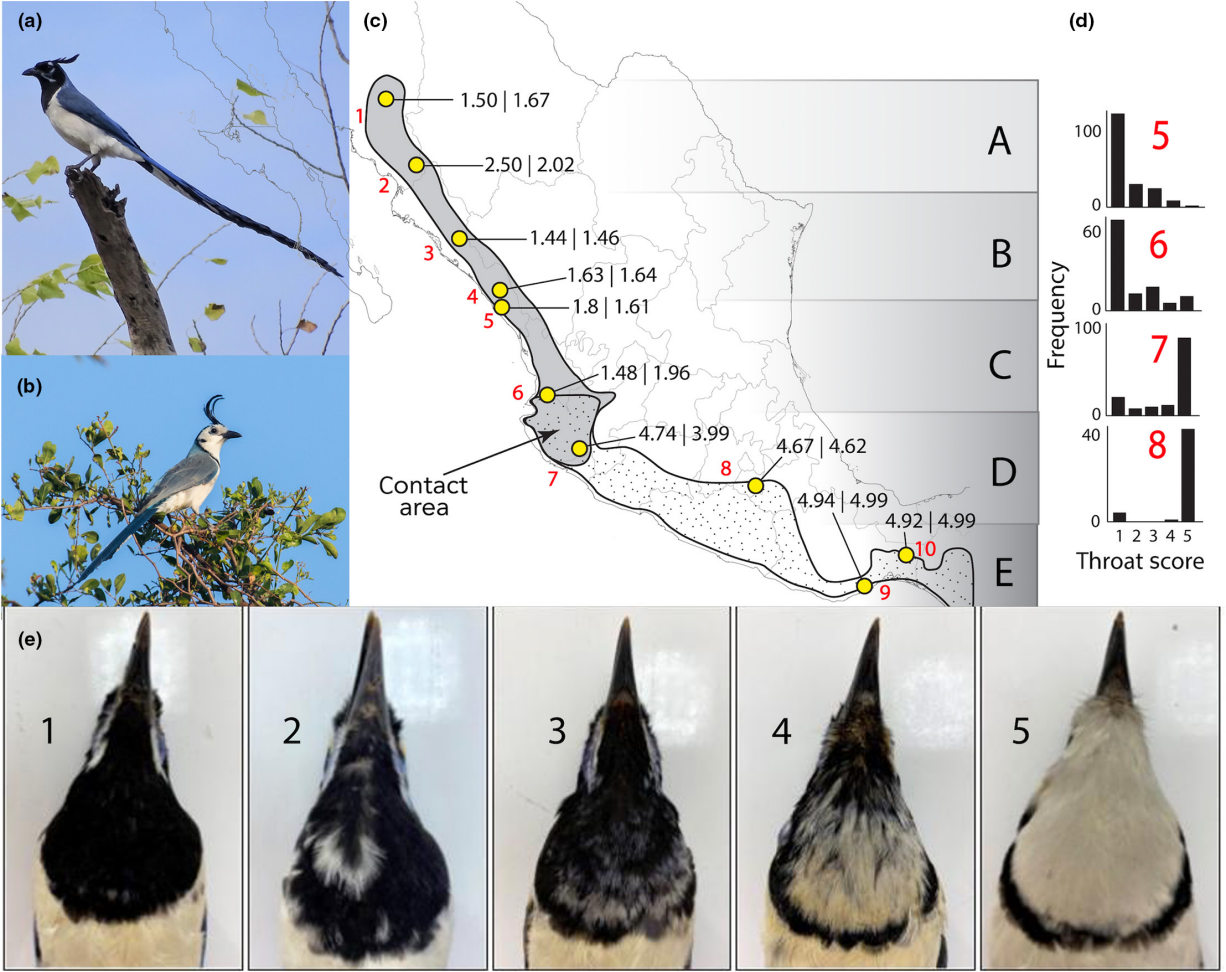
(a) Black‐throated magpie‐jay (*C. colliei*) from Culiacán, Sinaloa, Mexico, photo: Juan Ramón Manjarrez (iNaturalist 68514766 CC‐BY‐NC license); (b) white‐throated magpie‐jay (*C. formosa*) from Tehuantepec, Oaxaca, Mexico, photo: Liam Wolff (iNaturalist 177100291 CC‐BY‐NC license); (c) ranges of the two species, five geographical zones of comparison, and 10 sites for cline analysis with average throat scores in our historical (left) and modern (right) datasets; (d) transition in throat scores in the four populations nearest to the contact area; and (e) example specimens representing each of the categorical throat scores.

The black‐throated magpie‐jay (*Calocitta colliei*) and white‐throated magpie‐jay (*Calocitta formosa*) offer an ideal test case for using museum specimens and citizen science photos to assess temporal change in a hybrid zone. The two species are easily distinguishable in photos (Figure 1a,b). The black‐throated magpie‐jay ranges from Sinaloa in northwestern Mexico to the west coast of Jalisco, where it meets the range of the white‐throated magpie‐jay, which stretches southward through southern Mexico to Costa Rica (Figure 1c). Both species are highly ornamented with crests and long tails, but the black‐throated magpie‐jay has a longer tail and a black throat. The existence of hybrids where the ranges of the two species overlap in Jalisco has been somewhat controversial (summarized in Sánchez‐González et al., 2021). Some authors believe putative hybrids might simply be juvenile black‐throated magpie‐jays that have not attained fully melanized throats (Phillips, 1986). Yet, individuals of mixed phenotypes are continually reported in the area of range overlap (dos Anjos & de Juana, 2020). A recent genetic study of a handful of nuclear and mitochondrial loci added fuel to the controversy, finding that initial divergence between the species occurred over 3 million years ago and suggesting little evidence for modern gene flow (Sánchez‐González et al., 2021). In theory, hybrids should be distinguishable from juvenile black‐throated magpie‐jays by other plumage features like the lack of a fully melanized crest, duller blue feathers, and a yellow gape (dos Anjos & de Juana, 2020), offering a chance to evaluate these competing hypotheses via specimens and photos.

We combined historical museum specimens with modern photo‐vouchers to assess the phenotypic evidence for the hypothesized *Calocitta* hybrid zone and, if supported, test for change in its location and width between two time periods (pre‐1973 and post‐2005). The western lowlands of Mexico have experienced significant habitat alteration, especially after the 1970s when expansion of year‐round irrigation directly led to a shift in the proportion of mesic habitat from 5% to over 60% (Rohwer et al., 2015). As a result, some species native to more arid thorn scrub have declined, while other species have expanded or increased their abundance (Rohwer et al., 2015). Documenting the historical trajectory of the interface between *Calocitta* species will increase our understanding of the power of habitat modification to affect the outcomes of speciation and secondary contact.

## 2 METHODS

We used a categorical scale for quantifying throat color that was repeatable and well‐suited to both museum specimens and photos. We scored throats from 1 (black) to 5 (white), with scores of 2–4 given to birds with a mix of black and white feathers (Figure 1e). Because black‐throated magpie‐jays develop their black throats over their first year of life (dos Anjos & de Juana, 2020), we excluded juveniles through a variety of other plumage features including duller and more rounded blue feathers on the wings, shorter tails and crests, a yellow gape (for especially young birds), and by the lack of a fully melanized head crest.

Our historical data were taken from 280 georeferenced museum specimens from western Mexico collected from 1902 to 1973. We used a wide range of historical dates to increase sample size because the most intense human habitat alterations along Mexico's Pacific Coast occurred after 1975 (Rohwer et al., 2015). For our modern dataset, we scored throats from 1463 research‐grade photos submitted to iNaturalist from 2005 to 2021. We did not assess geoprivacy level because the level of location masking (22 × 22 km) was typically much less than the finest scale of our analysis. We scored photos if the throat was fully visible and in good lighting and other plumage features could be used to assess juvenile status.

We analyzed our data in two ways: over broad geographic areas with traditional tests for differences in means and at finer scale at the level of populations with cline analysis. First, we calculated average throat scores across five geographical zones of the same size (Figure 1c). Because Shapiro and Levene tests indicated that throat scores violated normality assumptions of parametric tests, we conducted Kruskall–Wallis tests of difference in means between adjacent zones for both modern and historical data. Second, we also used the R software package HZAR (Derryberry et al., 2014) to plot historical and modern trait clines and compare cline centers and cline widths. We clumped historical and modern data into the same 10 localities down the western coast of Mexico (Figure 1c). The localities were chosen to be as equidistant as possible but were constrained by the sampling limitations of the museum data. Using HZAR, we tested three different nested cline models, specifically (1) fixed minimum and maximum values, not allowing exponential tails; (2) minimum and maximum values estimated as free parameters, not allowing fixed tails; and (3) minimum and maximum values estimated as free parameters, and both tails allowed to be exponential and estimated as free parameters. For each dataset (historical and modern), we ran all three models, each with an MCMC length = 1 M generations, visually assessed convergence, and determined the optimal model based on AICc score. The best‐performing model for the historical transect was model 1, while the best‐performing model for the modern transect was model 3. Differences in cline centers and widths were assessed via the 2‐log likelihood confidence intervals for each point estimate.

## 3 RESULTS

The overall percentage of individuals with mixed phenotypes (i.e., throat score of 2–4) was similar between the historical (13%, *n* = 280) and modern (14%, *n* = 1463) transects. In the historical transect, analyzing across five geographical zones revealed a single significant difference in throat scores between zones C and D, i.e., at the species transition zone (*p* < .001; Table 1; Figure 1c). For the modern transect, throat scores likewise differed significantly between zones C and D (*p* < .001), but there were also significant differences within species: a marginally significant difference between zones A and B (*p* = .054) in *C. colliei* and a highly significant difference between zones D and E (*p* < .001; Table 1) in *C. formosa*. Because white‐throated magpie‐jays found in Mexico City could be a recently introduced population, we reran the analyses removing these individuals from the D and E geographic zones, and the results across this transition were still significant (*p* < .001).

**TABLE 1:** Throat scores for modern and historical transects.

Population	Historical		Modern	
	Average	*n*	Average	*n*
1	1.50	2	1.67	3
2	2.50	4	2.02	43
3	1.44	9	1.46	26
4	1.63	16	1.64	130
5	1.80	10	1.61	174
6	1.48	21	1.96	116
7	4.74	50	3.99	138
8	4.67	18	4.62	45
9	4.94	113	4.99	215
10	4.92	37	4.99	573

**FIGURE 2:**
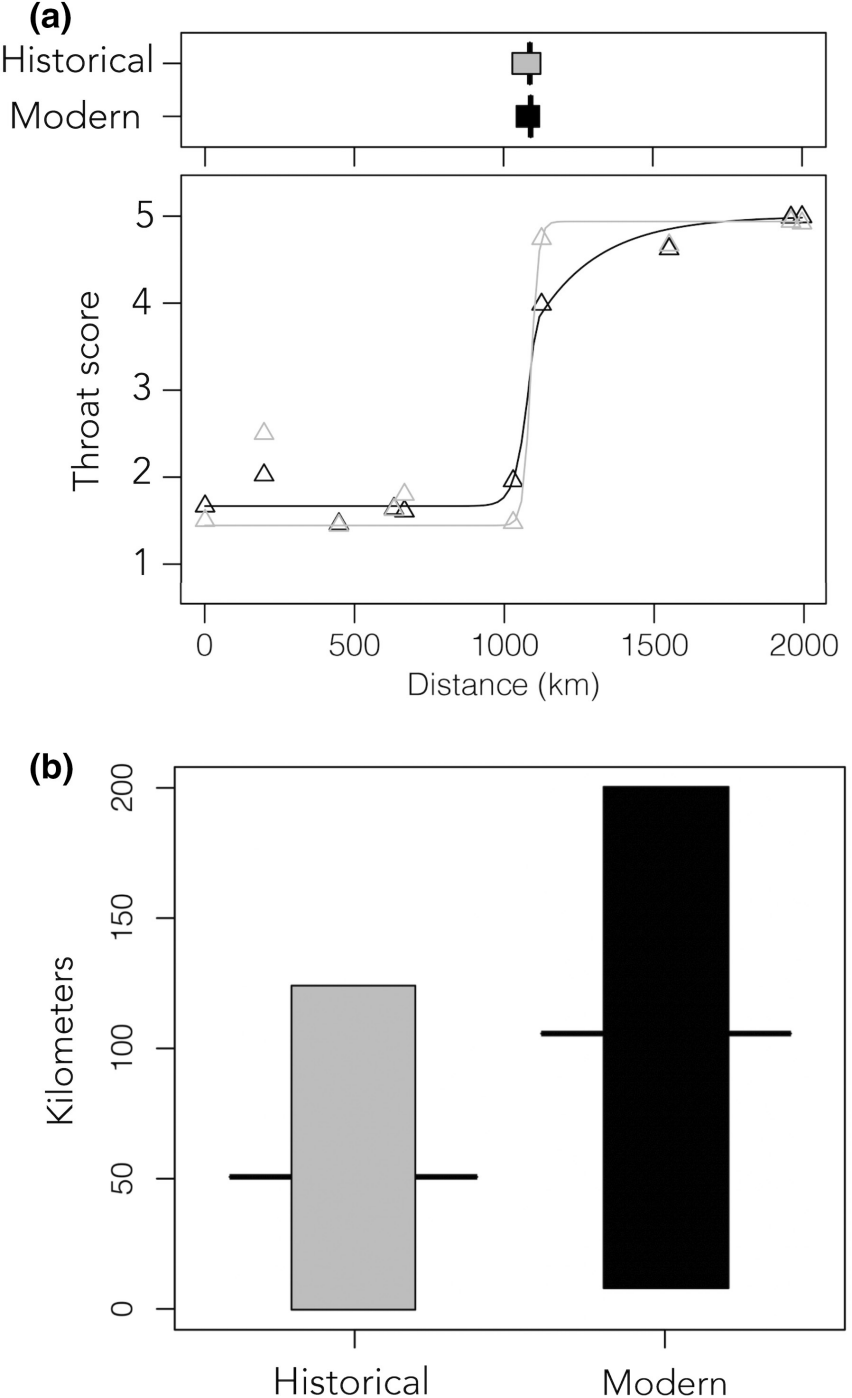
(a) Comparison of cline centers and geographic clines for historical (gray) and modern (black) data; (b) Comparison of cline widths. Bars represent the 2‐log likelihood error around each point estimate.

Analyzing the data in a cline framework also supported a sharp transition in the area of contact between sites 6 and 7 (Table 1; Figure 1c). The sigmoidal shape of the cline for the modern observation data was consistent with the expectation of a tension zone (Figure 2a), with increasing proportion of intermediate phenotypes approaching the cline center. For example, in the modern transect, the transition from populations 5 to 6 in the range of *C. colliei* was significant (Table 1; *p* = .037), with the population closer to the area of contact having a more intermediate phenotype. Similarly, in the range of *C. formosa*, population 7 had a significantly more intermediate phenotype than population 8 (Table 1; *p* = .023). The comparison of cline centers between modern and historical data suggests geographic stability of the hybrid zone between time periods (Figure 2a). The point estimates for cline width suggest that the modern cline is somewhat wider than the historical cline (100 km compared to 50 km), but the overlapping 2‐log likelihoods for these point estimates indicate that this difference is not statistically significant (Figure 2b).

## 4 DISCUSSION

Our results provide the first quantitative evidence for a hybrid zone between white‐throated and black‐throated magpie‐jays and further suggest that the hybrid zone has not changed substantially since the 1970s. The sigmoidal shape of the cline in the well‐sampled modern dataset is a hallmark of a hybrid zone (Barton & Hewitt, 1985) and is unlikely to be caused by sampling bias, intraspecific variation, or plumage development unrelated to introgression. While the elevated hybrid scores across the range of the black‐throated magpie‐jay (Table 1) might suggest that we could not remove all juveniles from the analysis, there is no reason why this would result in a sigmoidal cline shape with increasing number of intermediate throat types toward the cline center. And plumage development cannot explain the mirrored pattern of increasing intermediate phenotypes closer to the cline center in the range of the white‐throated magpie‐jay, which does not show age‐related effects in throat color.

Our results contrast with a recent genetic study of the *Calocitta* magpie‐jays, which concluded that modern gene flow in this system was unlikely, based on complete segregation of mitochondrial (mtDNA) haplotypes across the same transition area and monophyly of the two species in the mtDNA phylogeny (Sánchez‐González et al., 2021). However, it is worth noting that in avian systems, mtDNA regularly resists introgression due to processes like sex‐biased dispersal (Toews & Brelsford, 2012), mate choice (Lipshutz et al., 2019), and sex‐linked hybrid sterility (Carling & Brumfield, 2008; Gowen et al., 2014). Meanwhile, in nuclear markers, Sánchez‐González et al. (2021) uncovered shared alleles near the contact zone between these two species. For example, at the TGFB2 locus, distant parental populations are fixed for alternate alleles, while a population sampled from the middle of the contact zone displays a 50/50 mix of parental alleles—a pattern suggestive of ongoing gene flow. Further genomic study—including linked nuclear, mtDNA, and phenotypic data—will help disentangle signatures of gene flow from shared ancestral polymorphisms and allow investigators to test for differential introgression rates across markers and traits.

Our finding that the cline center for throat color has not changed dramatically in modern times provides a contrast to other temporal assessments of hybrid zones, which have often documented hybrid zone movement (Buggs, 2007; Wielstra, 2019), although notable cases of temporal stability have also been described (DeRaad et al., 2023; Wang et al., 2019). While the coarseness of our transect data (~200 km between sites on average) means that this hybrid zone might have shifted at smaller spatial scales than we can detect, the broad‐scale stability of this cline center is noteworthy given the amount of habitat conversion across the western Mexican lowlands since the 1970s. Very little of the original landscape of this region has been left intact, with scrub and thorn forests converted to agriculture and population centers on a massive scale. Habitat loss has created a patchier habitat matrix, associated with declines in some thorn forest species, while a shift to year‐round irrigation has also allowed other species to expand (Rohwer et al., 2015). It is easy to imagine either of these processes having a strong effect on the delicate balance of selection and gene flow in hybrid zone dynamics, yet our results indicate geographic stability over a period of at least 30 years. Future sampling between populations 6 and 7 in the heart of the cline center is needed. Unfortunately, this crucial area was not well collected in historical times; however, modern specimens and photos would provide more detail on the transition between species at a finer scale.

One puzzling pattern in both the historical and modern data is a pocket of white‐throated individuals in the northern part of the range of the black‐throated magpie‐jay (closest to site 2 of the transect). Here, the proportion of white throats increased from 28% to 33% from the historical to the modern time period. As mentioned above, it is possible that we were not able to entirely remove juvenile birds from our sampling, but this would not explain why there is a consistent geographic bias to this pattern unless the proportion of juvenile birds or developmental processes is somehow different at this site. One hypothesis that could explain this pattern is a human introduction of white‐throated magpie‐jays that pre‐dates the museum specimens we studied from the early 1900s. Magpie‐jays are often kept as pets, and introduced populations have popped up regularly across Mexico (Amador et al., 2009; Hernández‐Díaz et al., 2015) and the United States, including an introduced population of black‐throated magpie‐jays in Chula Vista, California (data from iNaturalist). Intriguingly, results from Sánchez‐González et al. (2021) show a distinctive nuclear allele near this more white‐throated population of black‐throated magpie‐jays, which is otherwise more common in the range of white‐throated magpie‐jays. Here again, genomic data linking genotype and phenotype could help solve this mystery, allowing us to distinguish whether these geographically anomalous white‐throated individuals are juveniles, mature birds bearing a novel mutation conferring a white throat, or admixed adult birds originating from a long‐distance introduction.

Finally, our study demonstrates how citizen science data associated with photo‐vouchers can be used to investigate evolutionary patterns such as temporal changes across zones of phenotypic transition. While discrete throat color differences offer a particularly amenable example, recent studies demonstrate that other more nuanced aspects of organismal coloration can also be quantified from citizen science photos (Hantak, Guralnick, Cameron, et al., 2022; Laitly et al., 2021), allowing for more varied study of hybrid zones and geographic variation in general. Photos are not replacements for museum specimens, as they capture only one axis of biological variation, whereas specimens allow study of internal anatomy, parasites, and genetics (Rocha et al., 2014), and open the door to future techniques that have not yet been developed. Still, the increasing geographic coverage of photographic databases offers a trove of information on geographic variation for the study of hybrid zones, speciation, and systematics (Leighton et al., 2016). iNaturalist has generally been used more for outreach and education (Aristeidou et al., 2021) and investigations of regional species diversity and species geographic ranges (Rosa et al., 2022) than for ecological and evolutionary studies, but this is now changing (Aguillon & Shultz, 2022; Bolt et al., 2022; Fritz & Ihlow, 2022; Putman et al., 2021). Analytical methods for tapping the full potential of citizen science photos (Hantak, Guralnick, Zare, et al., 2022; Leighton et al., 2016; Schiller et al., 2021), and better methods for imaging wildlife and specimens, including camera traps (Steenweg et al., 2017) and 3D models (Medina et al., 2020), will facilitate future studies bringing together photos and specimens in biodiversity science.

## AUTHOR CONTRIBUTIONS

**John E. McCormack:** Conceptualization (equal); funding acquisition (lead); formal analysis (supporting); writing – original draft (lead); writing – review and editing (equal). **Devon A. DeRaad:** Formal analysis (supporting); writing – review and editing (equal). **Alana K. Pizarro:** Conceptualization (equal); formal analysis (lead); writing – original draft (supporting); writing – review and editing (equal).

## CONFLICT OF INTEREST STATEMENT

The authors declare no competing interests.

## Supporting information


Appendix S1

## Data Availability

Raw data for throat scoring are available as Supporting Information. Code used for HZAR analysis is available at https://github.com/DevonDeRaad/magpie.jay.hzar.
